# GIS Walking Maps to Promote Physical Activity in Low-Income Public Housing Communities: a Qualitative Examination

**Published:** 2011-12-15

**Authors:** Lorna H. McNeill, Karen Emmons

**Affiliations:** Department of Health Disparities Research, University of Texas M.D. Anderson Cancer Center; Harvard School of Public Health and Dana-Farber Cancer Institute, Boston, Massachusetts

## Abstract

**Introduction:**

Walking is the most commonly reported leisure-time activity. Members of racial/ethnic minority groups and people of low socioeconomic status disproportionately live in urban environments that are perceived to be unsafe, thereby reducing opportunities for engaging in walking. We examined the use of walking maps for increasing physical activity (PA) among low-income residents of public housing sites in Boston, Massachusetts.

**Methods:**

PA facilities, local businesses, and destinations in a walkable half-mile radius of the housing community were identified and plotted on maps by using geographic information systems technology. Four focus groups (n = 24) were conducted to learn how the walking maps were used by the residents and to understand map features that promoted use.

**Results:**

Maps were used by participants to increase their PA, and use of the maps increased participants' awareness of community resources. Maps changed participants' perception of distances and were discussed as a means of fostering a sense of community. Use of the maps also increased participants' awareness of neighborhood incivilities. Barriers to map use were difficulty in interpreting the maps and lack of access to the maps.

**Conclusion:**

Walking maps that display PA opportunities and resources may be useful in increasing walking among residents of public housing sites.

## Introduction

Walking is the most commonly reported leisure-time physical activity (PA) (1,2) and is often reported as a primary activity among racial/ethnic minorities and people of low socioeconomic status (SES) (3,4). It is inexpensive, is convenient, and requires no formal equipment. For many people, walking is a necessity due to limited access to public transportation and low ownership of personal vehicles.

Evidence shows that the physical design of places where people live and work is associated with walking (5,6). Theoretically, urban environments are conducive to walking, given the abundance of sidewalks and parks and increased presence of public transportation, all of which are associated with walking (7,8). However, members of racial/ethnic minority groups and people of low SES disproportionately live in urban environments that are perceived to be unsafe, thereby reducing opportunities for engaging in walking (9). Interventions that simultaneously capitalize on the inherent PA resources in urban neighborhoods (eg, availability of sidewalks) and reduce the inherent barriers of such environments (eg, perceived danger) are needed.

Walking maps, which often include landmarks (eg, schools, streets) and walkable destinations (eg, popular businesses), are increasingly becoming popular PA facilitators (10) that may help effectively increase PA among underserved groups. Walking maps that identify and display nearby opportunities for PA and help members of racial/ethnic minority and low-income groups incorporate PA into their daily routines may reduce real and perceived barriers to PA. Maps that highlight parks and safe and convenient walking routes with marked distances can help people become more familiar with their neighborhoods and discover PA opportunities in walking distance from their homes. These maps may reduce common PA barriers (eg, safety perception) among members of racial/ethnic minority groups by helping them form positive perceptions of walking areas and providing an environmental cue to be active (11,12). Geographical information system (GIS) is a helpful tool for creating user-friendly maps. GIS maps spatially and accurately represent the physical characteristics of a neighborhood, such as streets, points of interest (ie, stores or museums), and natural resources (ie, lakes, green space, or parks).

Few studies have documented the use of maps to increase PA among groups (13) or have examined features of maps that may be helpful for residents of low-income communities. Many cities and states have created user-friendly maps to encourage their residents to be more physically active. However, few studies have evaluated the role of walking maps as a health promotion tool; therefore, we know little about the effectiveness of walking maps in increasing walking.

This article reports findings from focus groups conducted in 2006 with participants enrolled in Open Doors to Health, a randomized colorectal cancer prevention trial funded by the National Cancer Institute that was conducted from 2003 through 2008 and delivered to residents of low-income housing in Boston, Massachusetts. Details of the study have been described elsewhere (14). Walking maps were developed and piloted, and focus groups were conducted to understand map features that promoted use.

## Methods

### Walking map development

Maps were developed with substantial input from residents of housing sites, with emphasis on tailoring the maps to each housing site. On the basis of a literature review, we determined that the maps would include the 0.5-mile radius around each housing site as a walkable distance that would be achievable in approximately 30 minutes. One map was created for each housing site. A list of all potential walking destinations in that range was generated, which included parks and trails, businesses (eg, grocery stores, banks, post offices), and cultural destinations (eg, museums) (Figure). Although the primary route was 0.5 mile, we also included a 1-mile route to provide choice. Each walking destination was then identified using ArcGIS software (Esri, version 9.0, Redlands, California) and plotted on the maps. Maps included information about PA benefits and recommendations and information on safe walking practices that were specific to each neighborhood. User-friendly instructions helped residents to use the maps to plan a walk that could be completed in 30 minutes or less. Maps were created in both English and Spanish and were placed in high-traffic areas (eg, housing site lobby) and were distributed to participants through housing site residents who were recruited and trained to be peer leaders. Residents were also encouraged to use the walking maps to plan walks for the walking clubs that were organized as part of the intervention. Focus groups were held 3 to 6 months after the maps were created and distributed.

**Figure.**
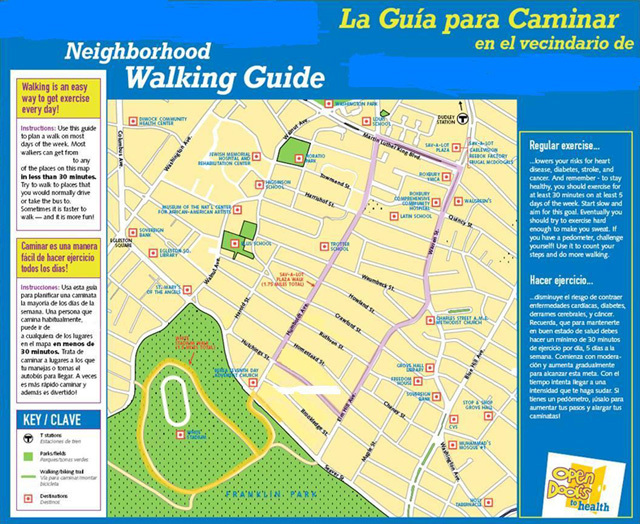
Example of a neighborhood walking guide distributed to focus group participants from Open Doors to Health, Boston, Massachusetts, 2006.

### Participants

Eligibility was residing in a participating housing site and being willing to participate. Participants were recruited by peer leaders and Open Doors to Health staff by word of mouth until a focus group session included 6 to 8 people. Each focus group was held at the housing site, and participants received a $20 gift card as an incentive to participate.

We selected 4 housing sites to participate in the focus groups — 3 that reportedly used the maps and 1 that did not. Residents of the housing sites included in this study were racially, ethnically, and demographically diverse. The first focus group was predominantly African American, the second was conducted with a Spanish-speaking Latino population and in Spanish, the third consisted of a housing site for the older people that had a mixed racial/ethnic population, and the fourth was conducted at the housing site of participants who did not use the map and was mixed by race/ethnicity and age.

### Procedure and analysis

Each focus group lasted approximately 1 hour and was conducted in English or Spanish, using a semistructured script with follow-up probes. Questions focused on how the walking maps were used for PA, how the maps influenced motivation for PA, features that participants liked and disliked about the maps, and barriers to and facilitators for using the maps.

All focus group interviews were recorded and transcribed verbatim by a professional transcriptionist. The Spanish transcript was first transcribed in Spanish and then translated to English. Two members of the research team reviewed transcripts and field notes to code transcripts. Coders met to resolve discrepancies and achieve consensus. An NVivo database (version 8, QSR International, Cambridge, Massachusetts) was created to enter codes first as free nodes and then as tree nodes to facilitate further analysis of the interviews. Major themes were determined a priori on the basis of the focus group interview guide. All interviews were systematically coded for emerging themes that were not specifically asked about. Quotes were selected to support explanation of themes. Themes presented reflect those most commonly mentioned by participants regarding how the walking maps were used for PA.

## Results

A total of 24 residents (3 men and 21 women) participated. Focus groups ranged in size from 4 to 13 participants per group (mean, 7 participants). Participants ranged in age from 20 to 65 years. Approximately 75% self-identified as black or African American, and almost all (94%) were born in the United States. Fifty-three percent reported having more than a high school education (primarily some college), 26% had high school/GED completion, and 21% had less than high school. Half of the participants reported working outside the home (31%, full-time; 19%, part-time), 11% self-identified as homemakers, and 39% indicated that they were unemployed (includes retired and disabled participants and homemakers).

Five key themes emerged from the focus group analysis: 1) increased awareness of neighborhood resources, 2) walking for pleasure and utilitarian purposes, 3) perception of distances, 4) community uses of the maps and fostering community, and 5) barriers to map use. Each of these themes is described below.

### Increased awareness of neighborhood resources

Participants used the maps for their traditional purpose: to learn where places are and how to get there. Although many participants were already familiar with many of the destinations, they used the maps to help them become more aware of their surroundings, discovering new places in their neighborhood and rediscovering other places. One participant stated,

. . . the more I looked into it . . . I did not know there were so many little things in the surrounding areas.

Very often, participants mixed talking about the walking guide with talking about a love of walking, which was partly inspired by participating in the intervention. Several participants mentioned discovering new things when walking or appreciating their neighborhood more through walking:

I liked seeing the growth of the neighborhood.

It makes you realize that we're in the middle, you know, of the heart of the city. You know, that? We're accessible to everything.

One Latino participant noted that she enjoyed using the map to walk because she discovered things that she had not seen in a long time:

Every time that one walks, one releases the mind . . . looking at the view, the [new] things . . . all the beauty.

Others talked about learning new information from the maps. For example, many participants learned by using the maps that 1 mile is equivalent to walking 4 laps around a standard running track.

Many participants were aware of existing incivilities in their neighborhood, such as garbage and graffiti, and for some residents the maps highlighted these negative aspects of their neighborhood. When using the maps to walk around their neighborhood, participants noticed how poorly maintained it was, having streets that needed to be cleaned up, broken street signs, and broken sidewalks. One participant, noting that the grass along a walking path was high and had not been cut in a while, stated

It should be much nicer kept . . . I don't think a bike path in any other town would be like that.

Others discovered new places that they had not seen. For example, when talking about using the map to walk to a new shopping center, a participant discovered that a new recreation area had been built:

It is beautiful and it has kind of like a little pool too, you know, and I find that it is [a] very interesting place and good [for] exercise . . . very beautiful.

### Walking for exercise and utilitarian walking

Focus group participants discussed places they walked to, including destinations that were on the map and others that were not. Participants were eager to talk about all the places they used the map to walk to: downtown, the post office, the drugstore, the supermarket, parks, the library, and community gardens. However, the most commonly reported use of the maps was for PA. Most focus group participants stated that they used the map to increase the amount of walking they did for exercise. Many residents already walked to get from place to place but used the maps to increase their intensity, speed, and motivation:

When you [start walking] first you just wanna get home, but still you want to know that accomplishment. That you accomplished something. That I did this and I walked.

Another participant used to live near a large park in another neighborhood and recently moved to the housing site. She used the guide to help her increase her activity in her new neighborhood.

I can walk in [the new neighborhood] without walking in the park and get the same results.

Several participants noted that they used the walking routes listed on the map in addition to discovering new walking routes. One female participant noted

Well we took advantage of the map because [now] we know how many miles we were walking. We walked around twice the same route that was on the map. For those that didn't want to walk too much. And for those who did wanna walk more, we did the same route over again.

### Perception of distances

There was consensus that walking outdoors helped residents become more familiar with their neighborhoods, particularly with respect to changing perception of distances. Several participants stated that, before using the maps, they perceived that taking the bus or driving was easier and faster than walking to many destinations. Several used the walking maps to see how far distances actually were and were quite surprised at how easy and fast it was to get to many of the destinations. For some participants, waiting on the bus could take up to 1 hour; they stated that they now prefer to walk:

I [used] to jump on the bus and go to Walgreens, and now that I know it was that close, I just walk.

I used to drive just to the post office, and that's ridiculous. Really! Now I'm just walking everywhere.

I used to take the bus to the [medical center] . . . I kinda walk now. It's a nice walk. I just [got] used to it.

Some focus group participants discussed how they would reach their destination more quickly walking than by driving:

Sometimes you can beat the bus walking. And you think you're doing a long walk, but if you look at the [map] it's not really that far.

### Community uses and fostering community

Several residents also mentioned that the walking map could be a resource for new residents of the neighborhood to help them become familiar with their new surroundings and highlight resources not well-known in the housing community. For example, 1 housing site had a community garden that focus group participants thought few residents were aware of; participants thought that including this information on the map would help new residents know about it.

Specific challenges related to using the maps in each community and ways to address these challenges were discussed. In 1 group, participants brainstormed ideas for distributing maps to new community residents. In particular, they thought the maps would help both newer and older residents learn about the positive history of the neighborhood and possibly encourage a sense of community. In another group, participants discussed the poor outdoor maintenance in their neighborhood and ways they could work together to improve the conditions.

### Barriers to map use

The most commonly cited barrier among participants of the focus group that was conducted with map nonusers (primarily older adults) was trouble interpreting the map; these participants found the walking map to be confusing and were unclear as to its purpose. Participants in this group, as well as those of the other groups, thought that one-on-one instruction on how to use the map was needed for many older residents.

Although maps were placed in central areas in each housing site (ie, outdoor, in the community center, near the doors, near the elevators), nonusers of the maps thought that additional maps should have been placed more prominently throughout the housing site to encourage use. A concern was that the highly trafficked areas where the maps were placed created more opportunities for vandalism, an issue that was emphasized by participants in the focus group of older residents.

The number of destinations on the map was mentioned as both a strength and a barrier. For some participants, the number of destinations, landmarks, and information included on the maps were just right. Other participants said they would have used the maps more if more and different destinations were indicated. Participants of only 1 focus group mentioned safety as a barrier to using the maps; these participants specifically suggested that we include a caution note that instructed map users to walk in groups for safety reasons.

## Discussion

Graphic displays of data can be powerful and informative, and walking maps that display PA opportunities and resources may be useful in increasing walking in the immediate neighborhood of urban, public housing residents. Our findings suggest that walking maps may be a tool for increasing walking in this setting. The maps capitalized on walking, the most commonly reported leisure-time activity, by making it more interesting and interactive. Focus group participants liked the maps and used them for their intended purpose: to increase their PA.

Studies indicate that more outdoor physical activity resources (ie, parks and green space) may be available in low-income neighborhoods than in high-income neighborhoods (15-17), and maps can provide a way for people to quickly identify these key features of their neighborhoods. However, resident perceptions of those resources are often poor (18). In our study, walking maps may have helped to create more positive neighborhood perceptions among participants, although the negative aspects of some neighborhoods may also have been reinforced.

The maps used in this study helped to reduce common psychosocial barriers to walking, such as lack of time and perceived safety. Focus group participants noted that walking was sometimes quicker than taking the bus to reach destinations, thereby potentially freeing up some of their time. Although neighborhood safety is often a concern among low-income and minority groups (19-21), participants of only 1 focus group mentioned safety as a barrier to using the maps. There may be several reasons for this finding. Many participants noted that they used the walking maps as part of their walking clubs and the walking clubs likely helped to increase feelings of safety. Furthermore, in the larger trial, 80% of participants reported their neighborhood to be safe in the daytime.

Our study has limitations. First, our study used data collected from focus group participants, which are subject to social acceptance bias, among other types of bias, in qualitative research. Second, we used a small sample size and participants from housing sites from 1 city, so our results may not be generalizable to residents of housing sites in other US cities. Third, several activities were simultaneously being implemented in the housing site during our study (eg, onsite exercise classes, peer support, walking clubs); therefore, the responses about the walking maps may be attributable to these other activities and not the maps.

We suggest recommendations for creating neighborhood walking maps. First, maps should be tailored to each neighborhood or area and should be created to be culturally appropriate, ideally with neighborhood resident input. Second, maps should be aesthetically pleasing but not too "busy." Achieving this characteristic may be challenging; a certain number of destinations should be included to promote use of the maps, but too many destinations make the maps hard to interpret. Third, the goal of the maps is to motivate residents to use them. Displaying maps in accessible places or providing maps to new residents may help to promote neighborhood walking or other collective activities.

*Healthy People 2020* goals for PA include increasing the proportion of trips made by walking (22). To achieve this national objective, innovative interventions and programs are needed that promote accessible and affordable community resources for PA and that simultaneously enhance the desirability of neighborhood pedestrian walking. Walking maps may be a tool for increasing walking, thereby addressing disparities in PA among underserved populations.
